# Serial Bronchoalveolar Lavage Fluid *Aspergillus* Galactomannan and Treatment Response in Invasive Pulmonary Aspergillosis

**DOI:** 10.1093/ofid/ofae114

**Published:** 2024-03-01

**Authors:** Daniel Z P Friedman, Elitza S Theel, Randall C Walker, Holenarasipur R Vikram, Raymund R Razonable, Paschalis Vergidis

**Affiliations:** Section of Infectious Disease, Department of Medicine, Mayo Clinic, Rochester, Minnesota, USA; Section of Infectious Diseases and Public Health, University of Chicago, Chicago, Illinois, USA; Division of Clinical Microbiology, Department of Laboratory Medicine and Pathology, Mayo Clinic, Rochester, Minnesota, USA; Section of Infectious Disease, Department of Medicine, Mayo Clinic, Rochester, Minnesota, USA; Division of Infectious Diseases, Mayo Clinic, Phoenix, Arizona, USA; Section of Infectious Disease, Department of Medicine, Mayo Clinic, Rochester, Minnesota, USA; William J von Liebig Center for Transplantation and Clinical Regeneration, Mayo Clinic, Rochester, Minnesota, USA; Section of Infectious Disease, Department of Medicine, Mayo Clinic, Rochester, Minnesota, USA; William J von Liebig Center for Transplantation and Clinical Regeneration, Mayo Clinic, Rochester, Minnesota, USA

**Keywords:** aspergillosis, bronchoscopy, galactomannan, hematopoietic stem cell transplant, solid organ transplant

## Abstract

We studied patients diagnosed with aspergillosis based on positive bronchoalveolar lavage (BAL) *Aspergillus* galactomannan (GM) who had follow-up BAL sampling within 180 days. GM trend and clinical outcome were concordant in only 60% (30/50). While useful for the initial diagnosis, BAL GM trending does not always correlate with treatment response.

Diagnosis of invasive pulmonary aspergillosis (IPA) remains challenging, especially when relying on conventional culture methods. Galactomannans (GMs) are polysaccharide components of the *Aspergillus* cell wall that can be detected in serum or bronchoalveolar lavage (BAL) fluid. In a meta-analysis of 50 studies, the sensitivity of serum *Aspergillus* GM was estimated at 82% [1]. The assay performs better in patients with hematologic malignancies and hematopoietic stem cell transplant recipients but has significantly lower sensitivity in non-neutropenic hosts [2]. Serial serum *Aspergillus* GM measurement has demonstrated clinical utility in monitoring high-risk individuals, as it may become positive before clinical or radiographic findings of aspergillosis. As such, serum *Aspergillus* GM has been used to guide preemptive therapy. In addition, changes in circulating serum *Aspergillus* GM have been shown to be predictive of clinical outcomes [3, 4].

When IPA is suspected in at-risk individuals with compatible clinical and radiographic findings, flexible bronchoscopy with BAL is commonly pursued to obtain specimens for diagnostic purposes. Using an optical density index (ODI) cutoff value of ≥0.5, the sensitivity of BAL *Aspergillus* GM has been estimated at 88% [5]. Although BAL *Aspergillus* GM positivity supports the diagnosis of IPA, the role of serial measurements in predicting clinical outcomes has not been studied. Our aim was to determine whether changes in BAL *Aspergillus* GM ODI correlate with response to antifungal therapy.

## METHODS

### Study Design, Patients, and Procedures

We performed a retrospective cohort study of patients diagnosed with IPA at Mayo Clinic sites (Rochester, MN, Phoenix, AZ, and Jacksonville, FL) between January 1, 2011, and January 31, 2021.

We included patients with a positive BAL *Aspergillus* GM at diagnosis who received mold-active antifungal therapy and had at least 1 follow-up BAL GM. The cohort consisted of patients with a follow-up GM from 7 to 180 days after diagnosis. A follow-up GM collected beyond 180 days was not considered to be associated with the original disease process.

### Patient Consent

The study was approved by the Mayo Clinic Institutional Review Board.

### Definitions and Outcomes

BAL GM was measured using the Platelia *Aspergillus* antigen immunoenzymatic assay (Bio-Rad Laboratories, Hercules, CA, USA) [6]. We considered the test to be positive if the ODI value was ≥0.5 based on the analytical threshold provided by the manufacturer. For patients who had >2 bronchoscopies during the study period, we included only the second ODI value.

IPA was categorized as proven or probable according to the European Organization for Research and Treatment of Cancer and Mycoses Study Group Education and Research Consortium (EORTC/MSGERC) consensus definitions [7]. We also used consensus definitions to characterize clinical response as success (complete response, partial response) or failure (stable response, progression of fungal disease, death) based on follow-up clinical and radiographic findings [8]. The date of follow-up imaging or death was defined as the clinical follow-up date. If >1 chest CT scan was performed, the first imaging study within 6–12 weeks after the diagnosis of IPA was selected to assess response.

BAL *Aspergillus* GM response was considered concordant with clinical response if a decline in GM was observed in a patient with successful clinical outcome or if an increase in GM (or stable GM) was observed in a patient with clinical failure.

### Statistical Analysis

Categorical variables were expressed as percentages and compared using the chi-square test or Fisher exact test, as appropriate. Continuous variables were expressed as mean (SD) or median (interquartile range [IQR]) according to their distribution and compared using the Student *t* test or Mann-Whitney test, respectively. ODI measurements ≥3.750 were assigned a value of 3.750 for the purposes of the analysis. A 2-sided *P* value <.05 was considered significant. All statistics were performed using Stata IC, version 16.

## RESULTS

We identified 218 patients with a positive BAL *Aspergillus* GM and at least 1 follow-up GM. We excluded 168 patients: 117 did not meet the criteria for IPA, 47 did not have a follow-up BAL GM within 7–180 days following diagnosis, and 4 had a concomitant fungal or untreated bacterial infection at follow-up bronchoscopy.

We studied 50 patients. The mean age at diagnosis was 60 years, and 70% were men. The most common underlying disease was immunosuppressive treatment in the 60 days preceding the diagnosis of IPA (96%), hematologic malignancy (58%), and severe neutropenia (54%). Eight patients (16%) had proven IPA, and the remaining 42 (84%) had probable IPA. All patients were started on antifungal treatment at the time of diagnosis of aspergillosis. Reasons for repeat bronchoscopy were radiographic progression (40%), new or worsening hypoxia (22%), fever (12%), airway secretion clearance (12%), post-transplant surveillance (10%), and hemoptysis (4%).

**Figure 1.**
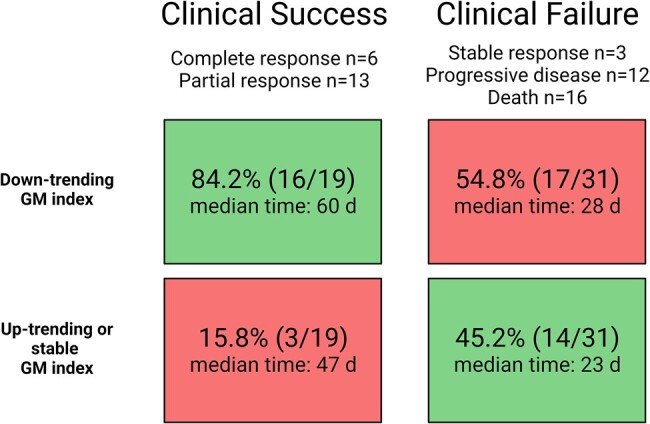
Clinical outcome in correlation to *Aspergillus* GM index trend (median time from collection of the initial to the follow-up bronchoscopic specimen). Abbreviation: GM, galactomannan.

The median GM index value at diagnosis was 3.680. Follow-up GM was collected at a median (IQR) of 32 (21–64) days after the initial collection, and clinical/radiographic follow-up occurred at a median (IQR) of 56 (36–80) days. The GM index trend in relation to clinical response is shown in Figure 1 and Supplementary Table 1.

Table 1 shows a comparison between those who had GM trends concordant to their clinical responses and those who had discordant findings. Concordant response was observed in 60% (30/50). A smaller decrease in median GM value (0.194 vs 2.321; *P* = .02) was associated with a concordant GM response. Almost half of patients (53.4%) with a concordant response had clinical and radiographic improvement, whereas only 13.0% of those with a discordant response had improvement (*P* < .01). Radiographic worsening could not be explained by a new infection or another pulmonary process. Patients with a solid organ malignancy were more likely to have a discordant GM response.

**Table 1.** Comparison Between Concordant and Discordant Results in Patients With Follow-up Bronchoscopy Within 7–180 Days After Diagnosis (n = 50)

Clinical Characteristic	Concordance (n = 30)	Discordance (n = 20)	*P* Value
Mean age (SD), y	59 (15)	61 (15)	.64
Male sex, No. (%)	22 (73.3)	13 (65.0)	.53
Disease classification, No. (%)			
Proven	7 (23.3)	1 (5.0)	.12
Probable	23 (76.7)	19 (95.0)	
Positive BAL culture for *Aspergillus* spp.^a^	14 (46.7)	9 (45.0)	.92
Median initial GM (IQR)	3.680 (1.488–3.750)	3.617 (1.676–3.750)	.66
Median time to follow-up GM (IQR), d	32 (23–64)	31 (20–62)	.67
Median GM decrease (IQR)	0.194 (0.000–1.968)	2.321 (0.711–3.250)	.02
Underlying disease, No. (%)			
Hematologic malignancy	17 (56.7)	12 (60.0)	.82
Allogeneic HSCT recipient	6 (20.0)	4 (20.0)	1.00
Graft-vs-host disease^b^	3 (10.0)	1 (5.0)	.64
SOT recipient	11 (36.7)	4 (20.0)	.21
Solid organ malignancy	0	4 (20.0)	.02
Structural lung disease	5 (16.7)	6 (30.0)	.31
Immunosuppressive treatment	29 (96.7)	19 (95.0)	1.00
Prolonged glucocorticoid use^c^	11 (36.7)	6 (30.0)	.62
Diabetes mellitus	8 (26.7)	4 (20.0)	.74
Severe neutropenia^d^	15 (50.0)	12 (60.0)	.49
Median duration of neutropenia (IQR), d	15 (8–18)	17 (7–35)	.51
Median time to outcome assessment (IQR), d	57 (35–82)	53 (37–79)	.67
Complete/partial response, No. (%)	16 (53.3)	3 (15.0)	<.01
Death, No. (%)	8 (26.7)	8 (40.0)	.32

Abbreviations: BAL, bronchoalveolar lavage fluid; GM, galactomannan; HSCT, hematopoietic stem cell transplant; IPA, invasive pulmonary aspergillosis; IQR, interquartile range; SD, standard deviation; SOT, solid organ transplant.

^a^ *Aspergillus* species: *A. fumigatus* (15), *A. terreus* (3), *A. niger* (3), *A. flavus* (2), *A. nidulans* (2), *A. versicolor* (2), *A. ustus* (1). More than 1 organism grew in 4 BAL samples.

^b^Graft-vs-host-disease, grade III/IV.

^c^Prolonged glucocorticoid use was defined as at least 15 mg/d of prednisone (or equivalent glucocorticoid dose) for at least 3 weeks in the preceding 60 days.

^d^Severe neutropenia was defined as absolute neutrophil count <0.5×10^9^/L in the preceding 60 days.

## DISCUSSION

Patients at risk for IPA often get repeated bronchoscopies for indications such as pulmonary hygiene, investigation of worsening pulmonary infiltrates, and routine surveillance following lung transplantation. During a 10-year period, we identified a large cohort of patients who had repeated BAL *Aspergillus* GM measurements. Our aim was to determine if there is a correlation between GM values and clinical outcome of IPA in patients who received antifungal therapy. The results of our study suggest that serial BAL *Aspergillus* GM is not a reliable marker in predicting treatment response. Nonetheless, we note that a stable or increasing BAL *Aspergillus* GM index was more commonly associated with clinical failure.

Lack of correlation between BAL *Aspergillus* GM trend and clinical outcomes may be related to lack of standardization in bronchoscopy including differences in technique. We note that there is variability between bronchoscopists or even between different procedures performed by the same bronchoscopist. Lavage is typically performed at the site where the disease is most prominent radiographically. The sites selected may differ between serial bronchoscopies. During the procedure, sterile saline is instilled and then fluid is recovered by suctioning. Several factors have been found to affect the recovery of BAL fluid [9]. Even with consistent volumes of saline administration for lavage, the amount of recovered fluid may vary. In technically challenging procedures due to stenosis or other anatomic abnormalities, fluid recovery may be significantly decreased, thus affecting *Aspergillus* GM concentration.

In the updated EORTC/MSGERC definitions of invasive fungal disease, a cutoff for positivity of BAL *Aspergillus* GM of ≥1.0 was adopted [10]. Although a higher GM index may increase the specificity of the assay [11], we opted to use the cutoff value of 0.5 (as suggested by the manufacturer and the US Food and Drug Administration). We note that only 7 out of 50 subjects had *Aspergillus* GM ODI between 0.5 and 1.0.

In our study, 4 patients diagnosed with solid organ malignancy had a decrease in BAL *Aspergillus* GM index despite clinical failure. Three of these patients had primary lung cancer or pulmonary metastasis; therefore, this discordance may be explained by the challenges in interpreting radiographic changes and selecting the lavage site in the background of underlying lung pathology.

Our study has inherent limitations due to its retrospective design. Most patients who underwent repeat bronchoscopy had worsening clinical or radiographic findings, which may have introduced a selection bias. We were not always able to determine if the airway location of BAL collection remained consistent between different bronchoscopies of the same patient. Serial bronchoscopies were not performed at regular intervals, and this may have impacted the trend of *Aspergillus* GM. Finally, the number of patients in the cohort does not allow for subgroup analysis based on the underlying immunocompromising condition.

Developing a surrogate outcome biomarker for IPA remains challenging due to the biological complexity of the disease, variable virulence of infecting *Aspergillus* species, host immune status, and timeliness of initiation of antifungal therapy [12]. In conclusion, we found that serial testing for *Aspergillus* GM in BAL does not consistently correlate with clinical and radiographic response of IPA and should not be routinely used to monitor treatment response.

## Supplementary Material

ofae114_Supplementary_Data

## Supplementary Data

Supplementary materials are available at *Open Forum Infectious Diseases* online. Consisting of data provided by the authors to benefit the reader, the posted materials are not copyedited and are the sole responsibility of the authors, so questions or comments should be addressed to the corresponding author.
