# Supplementary material for: Serial Bronchoalveolar Lavage Fluid Aspergillus Galactomannan and Treatment Response in Invasive Pulmonary Aspergillosis
Source: Open Forum Infect Dis. 2024 Mar 1;11(4):ofae114. doi: 10.1093/ofid/ofae114 (PMC10977621; doi:10.1093/ofid/ofae114)
Supplement: ofae114_Supplementary_Data [file ofae114_supplementary_data.docx]

**Supplementary Table 1.** Clinical Success/Failure and Aspergillus Galactomannan (GM) Index Trend in Patients with Follow-Up Bronchoscopy within 7-180 Days After Diagnosis.

| Clinical Characteristic | Clinical Success  Downtrending GM  (n=16) | Clinical Success  Uptrending/Stable GM  (n=3) | Clinical Failure  Downtrending GM  (n=17) | Clinical Failure  Uptrending/Stable GM  (n=14) |
| --- | --- | --- | --- | --- |
| Mean age, years (SD) | 54 (15.7) | 39 (28.9) | 65 (6.1) | 65 (12.5) |
| Male sex, n (%) | 13 (81.3) | 3 (100.0) | 10 (58..8) | 9 (64.3) |
| Disease Classification, n(%) |  | | | |
| Proven | 2 (12.5) | 0 | 1 (5.9) | 5 (35.7) |
| Probable | 14 (87.5) | 3 (100.0) | 16 (9.4) | 9 (64.2) |
| Positive BAL culture for *Aspergillus* spp. | 8 (50.0) | 2 (66.7) | 7 (41.2) | 6 (42.9) |
| Median initial GM (IQR) | 2.991 (1.405-3.750) | 2.647 (1.781-3.199) | 3.000 (1.716-3.750) | 3.716 (1.698-3.750) |
| Median time to follow-up GM, days (IQR) | 60 (32-97) | 47 (34-60) | 28 (16-62) | 23 (14-29) |
| Median GM change (IQR) | -1.913 (-0.905; -3.195) | 1.073 (0.537-1.088) | -2.941 (-1.117; -3.250) | 0 (0-0.410) |
| Underlying disease, n (%) |  | | | |
| Hematologic malignancy | 6 (37.5) | 2 (66.7) | 10 (58.8) | 11 (78.6) |
| Allogeneic HSCT recipient | 2 (12.5) | 1 (33.3) | 3 (17.6) | 4 (28.6) |
| Graft-versus-host disease *^a^* | 1 (6.3) | 0 | 1 (5.9) | 2 (14.3) |
| SOT recipient | 9 (56.3) | 0 | 4 (23.5) | 2 (14.3) |
| Solid organ malignancy | 0 | 0 | 4 (23.5) | 0 |
| Structural lung disease | 3 (18.8) | 0 | 6 (35.3) | 2 (14.3) |
| Immunosuppressive treatment | 15 (93.8) | 3 (100.0) | 16 (94.1) | 14 (100.0) |
| Prolonged glucocorticoid use *^b^* | 8 (50.0) | 2 (66.7) | 4 (23.5) | 3 (21.4) |
| Diabetes mellitus | 5 (31.3) | 0 | 4 (23.5) | 3 (21.4) |
| Severe neutropenia *^c^* | 5 (31.3) | 2 (66.7) | 10 (58.8) | 10 (71.4) |
| Median time to outcome assessment,  days (IQR) | 80 (62-102) | 74 (57-88) | 51 (35-79) | 38 (24-52) |
| Complete/Partial Response, n (%) | 16 (100.0) | 3 (100.0) | 0 | 0 |
| Death, n (%) | 0 | 0 | 8 (47.1) | 8 (57.1) |

Abbreviations: BAL, bronchoalveolar lavage fluid; GM, galactomannan; HSCT, hematopoietic stem cell transplant; IPA, invasive pulmonary aspergillosis; IQR, interquartile range; SD, standard deviation; SOT, solid organ transplant

*^a^*Graft-versus-host-disease, grade III/IV

*^b^*Prolonged glucocorticoid use was defined as at least 15 mg/day of prednisone (or equivalent dose) for at least three weeks in the preceding 60 days.

*^c^*Severe neutropenia was defined as absolute neutrophil count <0.5‧10^9^/L at any time in the preceding 60 days.
